# Prevalence and risk factors of oral mucositis in paediatric patients undergoing haematopoietic stem cell transplantation

**DOI:** 10.1111/odi.13777

**Published:** 2021-02-02

**Authors:** Abdulmalik Alhussain, Zikra Alkhayal, Mouhab Ayas, Hassan Abed

**Affiliations:** ^1^ North of Riyadh Dental Centre Central Second Health Cluster Riyadh Saudi Arabia; ^2^ Department of Dentistry King Faisal Specialist Hospital and Research Centre Riyadh Saudi Arabia; ^3^ Department of Paediatric Haematology/Oncology King Faisal Specialist Hospital and Research Centre Riyadh Saudi Arabia; ^4^ Department of Basic and Clinical Oral Sciences Faculty of Dentistry Umm Al‐Qura University Makkah Saudi Arabia

**Keywords:** chemotherapy, haematopoietic stem cell transplantation, oral hygiene, oral mucositis, paediatric patients, special care dentistry

## Abstract

**Background:**

A complete understanding of oral mucositis (OM) is crucial to develop appropriate interventions to aid in the successful overall health outcome of paediatric patients undergoing haematopoietic stem cell transplantation (HSCT).

**Aims:**

This study aimed at determining the prevalence and severity of OM and at identifying the predictive factors that might aggravate OM at one‐week, two‐week and three‐week post‐HSCT.

**Methods:**

This retrospective, hospital‐based study reviewed the medical records of 170 paediatric patients, summarising the patients’ characteristics using descriptive statistics. Binary logistic regression was used to identify factors associated with the development of OM.

**Results:**

At one‐week post‐HSCT, 41% of 140 patients (*n* = 49) had developed OM, this was reduced at two‐week (*n* = 36, 33%) and three‐week (*n* = 13, 19%) post‐HSCT. Univariate logistic regression revealed that patients with cancer (OR = 0.16, 95% CI = 0.05–0.54; *p*‐value = .003) had a significantly lower prevalence of OM. Younger patients with an average age of 7.9 years old (OR = 0.85, 95% CI = 0.75–0.97; *p*‐value = 0.013) and the presence of GvHD (OR = 2.37, 95% CI = 1.03–5.45, *p*‐value = 0.042) were significantly related to a higher prevalence of OM. Multivariable logistic regression confirmed that the risk of OM is lower in patients with cancer compared to those with immunodeficiency syndromes or hereditary blood diseases (OR = 0.18, 95% CI = 0.04–0.77; *p*‐value = .021).

**Conclusions:**

This study identified a significantly lower prevalence of OM in patients with cancer compared to other conditions and that young recipients and those who developed GvHD were more likely to have OM.

## 1 INTRODUCTION

Mucositis, or mucosal barrier injury, is a condition needing supportive care characterised by erythema, atrophy and ulceration of mucous membrane anywhere along the alimentary tract as a result of cancer therapy (Sonis, 2004). Oral mucositis (OM) is frequently occurring treatment‐induced side effect in patients with oncological and haematological disorders (Berger et al., 2018; Abed et al., 2019; Mubaraki et al., 2020). Haematopoietic stem cell transplantation (HSCT) is the treatment of choice for various malignancies, immune deficiencies and bone marrow failure syndromes in children, adolescents and adults (Barriga et al., 2012; Copelan, 2006). OM is arguably the single most painful and debilitating complication in patients undergoing high‐dose chemotherapy preparatory to HSCT (Bellm et al., 2000), with the incidence of OM among patients receiving both autologous and allogeneic HSCT varying between 75% and 99%, though OM tends to be more prevalent among paediatric patients (Eduardo et al., 2015; Vagliano et al., 2011).

OM is a predisposing factor for the development of further adverse implications, including an increased risk for local and systemic infections, poor nutrition and prolonged hospitalisation, as well as periodontal diseases, dental caries, dry mouth, trismus, dysphagia and dysgeusia (Mubaraki, 2019). Consequently, OM has a marked impact on the quality of life and cost of treatment (Mubaraki, 2019). The most invalidating complications of OM are encountered during the conditioning regimen prior to HSCT (Cinausero et al., 2017), and depending on the severity, OM might necessitate the interruption and/or dose reduction of anticancer therapy (Berger et al., 2018). However, little is known about the relevant factors associated with increased susceptibility of individuals to severe OM, specifically following HSCT (Bowen & Wardill, 2017). Complete healing of OM lesions typically occurs without scar formation unless it is exacerbated by a severe infection (Köstler et al., 2001).

Pathogenesis of OM is characterised by a complex five‐step biological process beginning with initiation, followed by primary damage response, signal amplification and eventually ulceration and healing (Sonis, 2004). OM is influenced by several risk factors which are usually classified into two main categories: patient‐related (i.e. age and type of malignancy) and treatment‐related (i.e. total body irradiation and type of cytotoxic agent) (Barasch & Peterson, 2003). Nevertheless, some prophylactic measures have been identified to minimise the intensity of OM (i.e. keratinocyte growth factor and cryotherapy) with currently no universally validated methods for prophylactic or therapeutic measures in children (Bowen & Wardill, 2017; Worthington et al., 2011). Also, the administration of opioid analgesics is often required to manage moderate to severe pain in children (Kuiken et al., 2015). Therefore, a complete understanding of this condition is crucial to develop appropriate interventions to aid in the successful overall health outcome. Given the scarce data available regarding OM in paediatric patients undergoing HSCT, this retrospective, hospital‐based study aimed at determining the prevalence and severity of OM, and at identifying the predictive factors that might aggravate OM at one‐week, two‐week and three‐week post‐HSCT.

## 2 MATERIALS AND METHODS

### 2.1 Ethical approval

Ethical approval was granted from the Institutional Review Board of the King Faisal Specialist Hospital and Research Centre (KFSH&RC), Riyadh, Saudi Arabia (RAC number: 2091015) before commencing any study‐related procedures.

### 2.2 Study design and setting

This was a retrospective analysis of the medical records of paediatric patients treated with HSCT at the Department of Haematology‐Oncology and Stem Cell Transplantation, KFSH&RC, Riyadh, Saudi Arabia.

### 2.3 Inclusion and exclusion criteria

Paediatric patients (0 to ≤ 14 years old) who received either autologous or allogeneic HSCT for the treatment of cancer (i.e. acute lymphocytic leukaemia, acute myelocytic leukaemia and solid tumours), hereditary blood disease (i.e. sickle cell anaemia, Fanconi anaemia and thalassaemia) and immune deficiency syndromes were eligible for inclusion in this study. Patients who had an oral health assessment for OM post‐HSCT were included in this study. Patients above 14 years of age and those who did not receive HSCT as part of the treatment or did not have oral health assessment for OM post‐HSCT were excluded from the study.

### 2.4 Procedure

The medical records of all patients (*n* = 170) who received HSCT in the study period were reviewed by the direct care team from the Dental Department at KFSH&RC, Riyadh, Saudi Arabia. As part of standard care in this study centre, all patients undergoing HSCT received instructions to use supersaturated calcium phosphate rinse and an extra‐soft toothbrush twice a day with their existing oral hygiene protocol regimen (0.2% chlorhexidine gluconate + 3% sodium bicarbonate + nystatin 100,000 U/ml). All patients received a unique ID (i.e. numbers from 1 to 170); thus, the patient's identity remained anonymous throughout the study, as the code number was only known to the direct care team. Patients’ demographic details (i.e. age and gender) and clinical parameters (i.e. medical condition, type of HSCT (i.e. allogeneic or autologous), presence of the graft versus host diseases (GvHD), presence and grade of OM and the average time between conditioning regimen administration and HSCT) and haematological parameters (i.e. white blood cells (WBCs), haemoglobin (HB) level and platelet count) were extracted from the patients’ medical records. All data were transferred into a Microsoft Excel spreadsheet. In this study, demographic and clinical/haematological parameters were recorded at three different times (i.e. one‐week, two‐week and three‐week post‐HSCT.

### 2.5 Measures

A limited set of well‐trained staff nurses was responsible for the assessment and documentation of OM, which was graded using the World Health Organization (WHO) oral toxicity fifth‐grade scale (WHO, 1979). The severity of OM was graded as follows: Grade 0 = solid diet, no soreness, no erythema, no ulcers, Grade I = solid diet, soreness and/or erythema, Grade II = solid diet, erythema and/or ulcers, Grade III = liquid diet, erythema and/or ulcers, and lastly Grade IV = unable to swallow, erythema and/or ulcers. Utilisation of this scale ensured ease of use, validity and reliability.

### 2.6 Statistical analysis

A descriptive analysis was performed to define the characteristics of the study sample through a form of counts and percentages. The normality of the data was checked using a histogram, Kolmogorov–Smirnov Lillefors and Levene's tests. A chi‐square test was used to establish a relationship between categorical variables. Accordingly, haematological parameters were compared between males and females using a parametric test (i.e. independent *t* tests), while the non‐parametric Kruskal–Wallis test was used to compare haematological parameters and medical conditions (i.e. cancer, hereditary blood diseases and immune deficiency syndromes). Binary logistic regression analysis was applied to identify factors associated with the occurrence of OM at univariate and multivariable levels. The statistical significance was assumed at a 5% level, and the statistical analysis was performed using the Statistical Package for Social Science (Released 2015, IBM SPSS Statistical for Windows, Version 23.0, Armonk, NY: IBM Corp).

### 2.7 Sample size

A minimum sample of 143 was enough to assess whether there is a significant difference in the prevalence of OM at three different times (i.e. one‐week, two‐week and three‐week post‐HSCT). The sample size was calculated based on *X* ^2^ tests (Goodness‐of‐fit tests: Contingency tables) at the 5% level of significance (α err prob = 0.05) with 80% power (1‐β err prob = 0.80), medium effect size (effect size w = 0.30), and Df of 5. The G*power 3.1.9.2 was used to calculate the sample size.

## 3 RESULTS

### 3.1 Sample characteristics

Of the 170 individuals, data of 140 eligible patients were included in the study analysis. An overview of their demographic characteristics is provided in Table 1. The average age was 8.8 years old (*SD* = 3.47), and most patients (*n* = 76, 54%) were male. In terms of medical conditions, some had been diagnosed with cancer (*n* = 35, 25%), and others had been diagnosed with hereditary blood diseases (*n* = 32, 23%), with most (*n* = 73, 52%) diagnosed with immunodeficiency syndromes. The vast majority of patients had allogeneic HSCT (*n* = 134, 96%) and did not develop GvHD (*n* = 93, 66%), with an average time between conditioning regimen administration and HSCT of 7.0 days (*SD* = 2.88).

**Table 1:** Demographic characteristics of the patients

Characteristics	Total (*n* = 140)		Male (*n* = 76)		Female (*n* = 64)	
	*n*	%	*n*	%	*n*	%
Age (mean; years)	8.8; *SD* = 3.47		8.9; *SD* = 3.59		8.8; *SD* = 3.36	
Medical condition						
Cancer	35	25.0	11	14.5	24	37.5
Hereditary blood diseases	32	22.9	21	27.6	11	17.2
Immunodeficiency syndromes	73	52.1	44	57.9	29	45.3
Type of HSCT						
Allogeneic	134	95.7	74	97.4	60	93.7
Autologous	6	4.3	2	2.6	4	6.3
GvHD						
No	93	66.4	49	64.5	44	68.7
Yes	47	33.6	27	35.5	20	31.3
Average time between conditioning regimen administration and HSCT (mean; days)	7.0; *SD* = 2.88		6.84; *SD* = 3.09		7.2; *SD* = 2.63	

### 3.2 Haematological parameters

Table 2 compares the haematological parameters of patients according to gender. Independent *t* test showed that there were no significant differences between males and females regarding the WBCs, HB level and platelets count at one‐week, two‐week and three‐week post‐HSCT (*p*‐value > .05).

**Table 2:** Haematological parameters of the patients according to gender (*n* = 140, unless otherwise stated)

Parameter
	Total	Male	Female	Statistical analysis	
	Mean; *SD*	Mean; *SD*	Mean; *SD*	*t*	*p*‐value
White blood cells count					
One‐week post‐HSCT (*n* = 131)	1.1; *SD* = 6.92	1.5; *SD* = 9.28	0.7; *SD* = 2.38	0.619	.546
Two‐week post‐HSCT (*n* = 130)	3.1; *SD* = 4.69	3.4; *SD* = 5.29	2.7; *SD* = 3.98	0.886	.377
Three‐week post‐HSCT (*n* = 105)	5.9; *SD* = 6.37	5.6; *SD* = 4.87	6.3; *SD* = 7.72	−0.559	.577
Haemoglobin level					
One‐week post‐HSCT (*n* = 131)	9.3; *SD* = 1.79	9.2; *SD* = 1.82	9.4; *SD* = 1.76	−0.759	.449
Two‐week post‐HSCT (*n* = 129)	9.4; *SD* = 1.29	9.5; *SD* = 1.48	9.4; *SD* = 1.03	0.510	.611
Three‐week post‐HSCT (*n* = 105)	9.5; *SD* = 1.54	9.4; *SD* = 1.50	9.5; *SD* = 1.59	−0.347	.729
Platelets count					
One‐week post‐HSCT (*n* = 130)	56.4; *SD* = 96.93	45.7; *SD* = 62.08	68.1; *SD* = 123.97	−1.319	.189
Two‐week post‐HSCT (*n* = 129)	47.5; *SD* = 81.40	51.7; *SD* = 81.62	42.5; *SD* = 81.49	0.659	.511
Three‐week post‐HSCT (*n* = 104)	48.6; *SD* = 56.60	43.3; *SD* = 46.91	54.5; *SD* = 65.89	−1.000	.319

Supporting File 1 shows the haematological parameters of patients according to medical conditions, with significant differences in WBCs count at two and three‐week post‐HSCT (H (*df* = 2) = 12.283, *p*‐value = .002, H (*df* = 2) = 7.935, *p*‐value = .019, respectively). Kruskal–Wallis test indicated that there were significant differences in platelets count at one‐ and three‐week post‐HSCT (H (*df* = 2) = 8.081, *p*‐value = .018, H (*df* = 2) = 10.702, *p*‐value = .005, respectively).

### 3.3 The prevalence of OM at the three different times (i.e. one‐week, two‐week and three‐week post‐HSCT)

Table 3 presents the prevalence and grade of OM. At one‐week post‐HSCT, 41% of patients (*n* = 49) developed OM, which reduced at two‐week (*n* = 36, 33%) and three‐week (*n* = 13, 19%) post‐HSCT.

**Table 3:** Oral mucositis details of the patients (*n* = 140, unless otherwise stated)

Parameter	None		Grade I		Grade II		Grade III		Grade IV	
	*n*	%	*n*	%	*n*	%	*n*	%	*n*	%
One‐week post‐HSCT (*n* = 120)	71	59.0	17	14.2	4	3.3	26	21.7	2	1.8
Two‐week post‐HSCT (*n* = 109)	73	67.0	9	8.2	0	0.0	21	19.3	6	5.5
Three‐week post‐HSCT (*n* = 69)	56	81.2	2	2.9	1	1.4	6	8.7	4	5.8

Table 4 presents OM development in relation to medical conditions, showing that the prevalence of OM was significantly higher at two‐week post‐HSCT among patients with immunodeficiency syndromes (*X* ^2^ = 11.200, *df* = 2, *p*‐value = .004).

**Table 4:** Oral mucositis development in relation to medical conditions

Parameter	Cancer (*n* = 35)		Hereditary blood diseases (*n* = 32)		Immunodeficiency syndromes (*n* = 73)		Statistical analysis	
	*n*	%	*n*	%	*n*	%	*X* ^2^ (*df* = 2)	*p*‐value
One‐week post‐HSCT (*n* = 49)	13	37.1	13	40.6	23	31.5	0.846	.655
Two‐week post‐HSCT (*n* = 36)	4	11.4	7	21.9	25	34.2	11.200	.004*
Three‐week post‐HSCT (*n* = 13)	0	0.0	4	12.5	9	12.3	4.105	.128

* *p*‐value < .01.

Table 5 presents OM development in relation to gender, showing a higher prevalence in male patients compared to females at one and two‐week post‐HSCT, with no statistically significant differences.

**Table 5:** Oral mucositis development in relation to gender

Parameter	Male (*n* = 76)		Female (*n* = 64)		Statistical analysis	
	*n*	%	*n*	%	*X* ^2^ (*df* = 1)	*p*‐value
One‐week post‐HSCT (*n* = 49)	30	39.5	19	29.7	1.662	.197
Two‐week post‐HSCT (*n* = 36)	20	26.3	16	25.0	0.004	.952
Three‐week post‐HSCT (*n* = 13)	7	9.2	6	9.4	0.112	.738

Supporting File 2 shows OM development in regard to the type of HSCT. More patients with allogeneic HSCT developed OM compared to those with autologous HSCT, but this was not statistically significant. However, because all patients received allogeneic HSCT at two‐ and three‐week post‐HSCT, no statistics computed to assess whether there is a significant difference.

### 3.4 Factors associated with the development of OM

#### 3.4.1 Univariate logistic regression analyses

Table 6 summarises the outcomes of binary logistic regression analysis for the factors associated with the development of OM, indicating that gender, type of HSCT, GvHD, the average time between conditioning regimen administration and HSCT, WBCs, HB levels and platelet count were not significantly related to the development of OM. Univariate logistic regression found that younger patients with an average age of 7.9 years old (*SD* = 3.61) had a significantly higher prevalence of OM at two‐week post‐HSCT compared to patients with an average age of 9.7 years old (*SD* = 3.27) (OR = 0.85, 95% CI = 0.75–0.97; *p*‐value = .013). Similarly, the presence of GvHD was significantly related to a higher prevalence of OM at two‐week post‐HSCT (OR = 2.37, 95% CI = 1.03–5.45, *p*‐value = .042). For example, 47% of patients who had GvHD developed OM versus 27%. Patients with cancer (*n* = 4, 11%) had significantly a lower prevalence of OM at two‐week post‐HSCT (OR = 0.16, 95% CI = 0.05–0.54; *p*‐value = .003).

**Table 6:** The outcomes of binary logistic regression analysis at univariate level for factors associated with OM

Characteristics	OM		“No OM”		B	SE	OR	95% CI	*p*‐value
One‐week post‐HSCT									
	(*n* = 49)		(*n* = 71)						
Age (mean; years)	8.7; *SD* = 3.57		9.0; *SD* = 3.30		−0.30	0.05	0.97	0.87–1.08	.580
	***n***	**%**	***n***	**%**					
Gender									
Male	30	61.2	35	49.3	‐	‐	1.00	Ref.	
Female	19	38.8	36	50.7	−0.48	0.38	0.62	0.29–1.29	.199
Medical condition									
Immunodeficiency syndromes	13	26.5	19	26.8	‐	‐	1.00	Ref.	
Cancer	13	26.5	14	19.7	0.12	0.45	1.13	0.47–2.71	.784
Hereditary blood diseases	23	47.0	38	53.5	0.43	0.47	1.53	0.61–3.83	.359
Type of HSCT									
Allogeneic	46	93.9	68	95.8	‐	‐	1.00	Ref.	
Autologous	3	6.1	3	4.2	0.39	0.84	1.48	0.29–7.65	.641
GvHD									
No	34	69.4	46	64.8	‐	‐	1.00	Ref.	
Yes	15	30.6	25	35.2	−0.21	0.39	0.81	0.37–1.77	.600
Average time between conditioning regimen administration and HSCT (mean/days)	7.2; *SD* = 3.19		6.7; *SD* = 2.73		0.06	0.06	1.06	0.93–1.20	.377
Haematological parameter									
White blood cells count	0.2; *SD* = 0.59		1.7; *SD* = 9.50		−0.38	0.33	0.68	0.36–1.31	.250
Haemoglobin level	9.2; *SD* = 1.96		9.3; *SD* = 1.75		−0.00	0.01	0.99	0.98–1.02	.684
Platelets count	33.8; *SD* = 38.80		55.1; *SD* = 72.01		−0.01	0.00	0.99	0.98–1.00	.078

Two‐week post‐HSCT									
	(*n* = 36)		(*n* = 73)						
Age (mean; years)	7.9; *SD* = 3.61		9.7; *SD* = 3.27		−0.16	0.06	0.85	0.75–0.97	.013
Gender									
Male	20	55.5	41	56.2	‐	‐	1.00	Ref.	
Female	16	44.4	32	43.8	0.02	0.41	1.02	0.46–2.29	.952
Medical condition									
Immunodeficiency syndromes	25	69.4	27	36.9	‐	‐	1.00	Ref.	
Cancer	4	11.1	26	35.6	−1.79	0.60	0.16	0.05–0.54	.003**
Hereditary blood diseases	7	19.5	20	27.5	−0.97	0.52	0.38	0.14–1.05	.061
Type of HSCT***									
Allogeneic	36	100.0	67	91.8	‐	‐	1.00	Ref.	
Autologous	0	0.0	6	8.2	‐	‐	‐	‐	.999
GvHD									
No	19	52.8	53	72.6	‐	‐	1.00	Ref.	
Yes	17	47.2	20	27.4	0.86	0.42	2.37	1.03–5.45	.042*
Average time between conditioning regimen administration and HSCT (mean; days)	7.0; *SD* = 2.64		7.1; *SD* = 3.26		−0.02	0.07	0.98	0.86–1.12	.774
Haematological parameter									
White blood cells count	2.0; *SD* = 3.85		3.3; *SD* = 5.28		−0.07	0.05	0.94	0.84–1.04	.222
Haemoglobin level	9.2;*SD* = 1.26		9.5; *SD* = 1.26		−0.02	0.02	0.98	0.95–1.01	.220
Platelets count	32.5;*SD* = 26.10		44.9; *SD* = 75.75		0.00	0.01	1.00	0.99–1.01	.382

Three‐week post‐HSCT									
	(*n* = 13)		(*n* = 56)						
Age (mean; years)	8.8; *SD* = 3.37		9.1; *SD* = 3.64		−0.02	0.09	0.98	0.82–1.16	.782
Gender									
Male	7	53.8	33	58.9	‐	‐	1.00	Ref.	
Female	6	46.2	23	41.1	0.21	0.62	1.23	0.36–4.14	.738
Medical condition									
Immunodeficiency syndromes	0	0.0	14	25.0	‐	‐	1.00	Ref.	
Cancer	4	30.8	12	21.4	‐	‐	‐	‐	.999
Hereditary blood diseases	9	69.2	30	53.6	0.11	0.69	1.11	0.29–4.31	.879
Type of HSCT									
Allogeneic	13	100.0	56	100.0	‐	‐	1.00	Ref.	
Autologous	0	0.0	0	0.0	‐	‐		‐	.999
GvHD									
No	10	76.9	30	53.6	‐	‐	1.00	Ref.	
Yes	3	23.1	26	46.4	−1.06	0.71	0.35	0.08–1.39	.136
Average time between conditioning regimen administration and HSCT (mean; days)	7.0; *SD* = 3.16		6.7; *SD* = 3.03		0.03	0.10	1.03	0.84–1.25	.788
Haematological parameter									
White blood cells count	3.6; *SD* = 5.73		6.4; *SD* = 7.32		−0.09	0.07	0.92	0.79–1.05	0.223
Haemoglobin level	8.9;*SD* = 1.82		9.4; *SD* = 1.24		−0.03	0.03	0.97	0.92–1.02	0.240
Platelets count	48.6;*SD* = 70.25		48.0; *SD* = 44.41		0.00	0.01	1.00	0.99–1.01	0.970

* *p*‐value < .05.

** *p*‐value < .01.

*** Insufficient numbers to run binary logistic regression analyses.

#### 3.4.2 Multivariable logistic regression analyses

Multivariable logistic regression confirmed that the risk of OM decreases in patients with cancer compared to those with immunodeficiency syndromes or hereditary blood diseases independently (OR = 0.18, 95% CI = 0.04–0.77; *p*‐value = .021)—see Table 7. Using direct entry, the Nagelkreke *R* ^2^ was 0.21, indicating that the variables predicted an estimated 21% of the variance in the development of OM.

**Table 7:** The outcomes of binary logistic regression analysis at multivariable level for factors associated with OM

	OM		“No OM”		B	SE	Odds ratio (OR)	95% CI	*p*‐value
One‐week post‐HSCT									
	(*n* = 49)		(*n* = 71)						
Age (mean; years)	8.7; *SD* = 3.57		9.0; *SD* = 3.30		−0.12	0.07	0.89	0.78–1.02	.077
	***n***	**%**	***n***	**%**					
Gender									
Male	30	61.2	35	49.3	‐	‐	1.00	Ref.	
Female	19	38.8	36	50.7	−0.77	0.42	0.46	0.20–1.06	.067
Medical condition									
Immunodeficiency syndromes	13	26.5	19	26.8	‐	‐	1.00	Ref.	
Cancer	13	26.5	14	19.7	−0.01	0.55	0.99	0.33–2.92	.983
Hereditary blood diseases	23	47.0	38	53.5	0.24	0.60	1.27	0.39–4.11	.691
Type of HSCT									
Allogeneic	46	93.9	68	95.8	‐	‐	1.00	Ref.	
Autologous	3	6.1	3	4.2	0.23	0.94	1.26	0.20–7.92	.804
GvHD									
No	34	69.4	46	64.8	‐	‐	1.00	Ref.	
Yes	15	30.6	25	35.2	−0.52	0.45	0.59	0.24–1.44	.249
Average time between conditioning regimen administration and HSCT (mean/days)	7.2; *SD* = 3.19		6.7; *SD* = 2.73		0.03	0.07	1.03	0.89–1.19	.675
Haematological parameter									
White blood cells count	0.2; *SD* = 0.59		1.7; *SD* = 9.50		−0.09	0.37	0.91	0.44–1.88	.803
Haemoglobin level	9.2; *SD* = 1.96	9.3; *SD* = 1.75	−0.01	0.01	0.99	0.97–1.02	0.550	0.97–1.02	.550
Platelets count	33.8; *SD* = 38.80	55.1; *SD* = 72.01	−0.01	0.01	0.99	0.98–1.00	0.192	0.98–1.00	.192

Two‐week post‐HSCT									
	(*n* = 36)		(*n* = 73)						
Age (mean; years)	7.9; *SD* = 3.61		9.7; *SD* = 3.27		−0.14	0.08	0.87	0.74–1.02	.079
Gender									
Male	20	55.5	41	56.2	‐	‐	1.00	Ref.	
Female	16	44.4	32	43.8	−0.04	0.50	0.96	0.36–2.59	.940
Medical condition									
Immunodeficiency syndromes	25	69.4	27	36.9	‐	‐	1.00	Ref.	
Cancer	4	11.1	26	35.6	1.68	0.73	0.18	0.04–0.77	.021*
Hereditary blood diseases	7	19.5	20	27.5	−0.99	0.71	0.37	0.09–1.48	.161
Type of HSCT**									
Allogeneic	36	100.0	67	91.8	‐	‐	1.00	Ref.	
Autologous	0	0.0	6	8.2	‐	‐	‐	‐	.999
GvHD									
No	19	52.8	53	72.6	‐	‐	1.00	Ref.	
Yes	17	47.2	20	27.4	0.75	0.51	2.12	0.79–5.71	.137
Average time between conditioning regimen administration and HSCT (mean/days)	7.0; *SD* = 2.64		7.1; *SD* = 3.26		0.07	0.09	1.07	0.89–1.29	.470
Haematological parameter									
White blood cells count	2.0; *SD* = 3.85		3.3; *SD* = 5.28		−0.05	0.07	0.95	0.82–1.09	.461
Haemoglobin level	9.2;*SD* = 1.26		9.5; *SD* = 1.26		−0.01	0.02	0.99	0.96–1.03	.723
Platelets count	32.5;*SD* = 26.10		44.9; *SD* = 75.75		−0.01	0.01	0.99	0.97–1.00	.386

Three‐week post‐HSCT									
	(*n* = 13)		(*n* = 56)						
Age (mean; years)	8.8; *SD* = 3.37		9.1; *SD* = 3.64		0.09	0.13	1.10	0.86–1.41	.452
Gender									
Male	7	53.8	33	58.9	‐	‐	1.00	Ref.	
Female	6	46.2	23	41.1	0.14	0.80	1.15	0.24–5.54	.863
Medical condition									
Immunodeficiency syndromes	0	0.0	14	25.0	‐	‐	1.00	Ref.	
Cancer	4	30.8	12	21.4	‐	‐	‐	‐	.999
Hereditary blood diseases	9	69.2	30	53.6	−0.54	1.29	0.58	0.05–7.27	.677
Type of HSCT									
Allogeneic	13	100.0	56	100.0	‐	‐	1.00	Ref.	
Autologous	0	0.0	0	0.0	‐	‐		‐	.999
GvHD									
No	10	76.9	30	53.6	‐	‐	1.00	Ref.	
Yes	3	23.1	26	46.4	−1.30	0.97	0.27	0.04–1.81	.179
Average time between conditioning regimen administration and HSCT (mean/days)	7.0; *SD* = 3.16		6.7; *SD* = 3.03		0.39	0.25	1.48	0.90–2.41	.121
Haematological parameter									
White blood cells count	3.6; *SD* = 5.73		6.4; *SD* = 7.32		−0.11	0.09	0.89	0.74–1.08	.247
Haemoglobin level	8.9;*SD* = 1.82		9.4; *SD* = 1.24		−0.03	0.04	0.97	0.90–1.04	.415
Platelets count	48.6;*SD* = 70.25		48.0; *SD* = 44.41		0.00	0.01	1.00	0.98–1.01	.746

* *p*‐value < .05.

** Insufficient numbers to run binary logistic regression analyses.

## 4 DISCUSSION

OM in paediatric patients constitutes a major oncological dilemma with practical limitations for assessment tools and treatment methods (Farrington & Cullen, 2010). Based on the recommendation of the literature to further investigate the risk prediction of OM, especially among the paediatric population (Bowen & Wardill, 2017), the current retrospective study aimed at determining the prevalence and severity of OM and at identifying the predictive factors that might aggravate OM early after HSCT infusion.

A recently published study pointed out that the incidence of OM among children (4–17 years) after HSCT (all grades of severity) was 80% (Kamsvåg et al., 2020). Similarly, a multi‐centre study of 262 children/adolescents aged between 0 to 18 years old reported that 79.8% developed OM (Vagliano et al., 2011). In contrast, the current study found that the prevalence of OM among paediatric patients (≤14 years) was 41%, making our findings inconsistent with those in the literature. This contradictory result might be due to the inability to assess the oral cavity properly in uncooperative and extremely young patients, as well as the incapacity of the child to attribute pain to bodily locations, or rather because of the prevention protocols that were implemented in the study centre, which we believe may have influenced the onset of OM, as those oral hygiene regimens were suggested to be of benefit in the reduction of OM occurrence (McGuire et al., 2013; Papas et al., 2003; American Academy of Paediatric Dentistry, 2013). The aforementioned challenges impose difficulties on the assessment and documentation of OM, which might contribute to missing data, which have been addressed in several studies (Bowen & Wardill, 2017; Jacobs et al., 2013; Tomlinson et al., 2008).

Regarding the analysis of OM severity according to the type of HSCT, our finding agreed with prior studies that observed no significant differences between patients receiving autologous or allogeneic HSCT (Kamsvåg et al., 2020; Vagliano et al., 2011). Similarly, we noticed that neither the incidence nor the severity of OM were significantly different between female and male patients, as reported previously in children who received HSCT or chemo‐ and radiotherapy (Carreón‐Burciaga et al., 2018).

The present study findings indicated that younger patients are significantly more prone to have OM at the two‐week post‐HSCT, similar to a previous report, wherein OM was found to be significantly influenced by the recipient age (Bardellini et al., 2013). A possible explanation for this might be linked to the high proliferative rate of basal epithelial cells in younger individuals (Sonis, 2007, 2011).

When the three underlying categories of medical conditions were compared, the prevalence of OM in the second‐week post‐HSCT was significantly lower in patients with cancer, whereas immunocompromised patients showed the greatest prevalence, which may be related to the type of conditioning regimen used rather than to the disease itself. For instance, a prospective evaluation study indicated that the principal determinant of OM was the preparative conditioning regimen (Wardley et al., 2000). Therefore, a powered, prospective study that focuses on the effect of the conditioning regimen with regard to the type of medical condition on the risk of OM development following HSCT is needed.

In our study, the presence of GvHD was significantly related to the development of OM. However, a retrospective study found no association between GvHD and OM, as they reported that the use of prophylaxis against GvHD was more likely to induce OM rather than GvHD itself (Bardellini et al., 2013). It is worth mentioning that Bardellini and his colleagues used only descriptive statistics; hence, they only assessed association, while our study used inferential statistics which assessed risk factors associated with OM. Furthermore, Bardellini et al. assessed risk factors for OM in paediatric patients who had only primary immunodeficiencies, not including other medical conditions such as cancer and hereditary blood diseases.

## 5 LIMITATIONS AND FUTURE RESEARCH

One of the limitations of the current study was the retrospective nature of the data analysis, which could have contributed to missing data. Additionally, this was a single‐centre study; hence this limits the generalisability of the results. Also, some potential confounding factors were not addressed in this study, which could influence OM development such as conditioning regimen, GvHD prophylaxis, nutritional route and whether or not the patients were hospitalised. Future studies may incorporate multiple centres to address the above‐mentioned variables to overcome the current limitations.

## 6 CONCLUSIONS AND RECOMMENDATIONS

Our study identified that young recipients and those who developed GvHD were more likely to have OM post‐HSCT. Moreover, the findings confirmed a significant decrease in the prevalence of OM in patients with cancer, raising the possibility that cancer patients might have obtained superior benefits of HSCT treatment relevant to their oral health compared to patients with other medical conditions who deserve equal or even greater attention. Therefore, comprehensive pre‐ and post‐HSCT oral care should also be emphasised for young patients with immunodeficiency syndromes or hereditary blood diseases. There is also a need for continued efforts directed to researching the risk prediction among this group of patients to develop intervention strategies and to establish evidence‐based guidelines for proper management of this condition.

## CONFLICT OF INTEREST

The authors declare no conflict of interest.

## AUTHOR CONTRIBUTIONS

**Abdulmalik Alhussain:** Data curation; Formal analysis; Methodology; Writing‐original draft; Writing‐review & editing. **Zikra Alkhayal:** Conceptualization; Funding acquisition; Project administration; Resources; Supervision. **Mouhab Ayas:** Conceptualization; Validation; Visualization. **Hassan Abed:** Conceptualization; Formal analysis; Methodology; Software; Writing‐original draft; Writing‐review & editing.

## ETHICAL APPROVAL

Ethical approval for this project was obtained from the Institutional Review Board of the King Faisal Specialist Hospital and Research Centre, Riyadh, Saudi Arabia (RAC number: 2091015).

### PEER REVIEW

The peer review history for this article is available at https://publons.com/publon/10.1111/odi.13777.

## Supporting information

Supplementary Material
