# Supplementary material for: Prevalence and risk factors of oral mucositis in paediatric patients undergoing haematopoietic stem cell transplantation
Source: Oral Dis. 2021 Feb 2;28(3):657–69. doi: 10.1111/odi.13777 (PMC9291549; doi:10.1111/odi.13777)
Supplement: Supplementary file 1 — Supplementary Material [file ODI-28-657-s001.docx]

| **Supporting File 1**: Haematological parameters of the patients according to medical condition. | | | | | | |
| --- | --- | --- | --- | --- | --- | --- |
| **Parameter** |  | **Cancer** | **HBD*** | **IDS**** | **Statistical analysis** | |
|  |  | **Median** | **Median** | **Median** | **H (df=2)***** | **P-value** |
| **White blood cells count** | One-week post-HSCT | 0.04 | 0.04 | 0.08 | 5.556 | 0.062 |
|  | Two-week post-HSCT | 0.9 | 2.3 | 0.7 | 12.283 | 0.002**** |
|  | Three-week post-HSCT | 4.1 | 5.4 | 3.1 | 7.935 | 0.019***** |
| **Haemoglobin level** | One-week post-HSCT | 9.3 | 8.8 | 9.1 | 0.950 | 0.622 |
|  | Two-week post-HSCT | 9.5 | 9.4 | 9.1 | 1.925 | 0.382 |
|  | Three-week post-HSCT | 9.4 | 9.5 | 9.0 | 3.493 | 0.174 |
| **Platelets count** | One-week post-HSCT | 19.0 | 23.0 | 33.0 | 8.081 | 0.018***** |
|  | Two-week post-HSCT | 23.3 | 25.0 | 28.0 | 0.628 | 0.731 |
|  | Three-week post-HSCT | 30.0 | 59.5 | 27.5 | 10.702 | 0.005**** |
| * HBD: Hereditary blood diseases; **IDS: Immunodeficiency syndromes, *** Kruskal Wallis test, ****P-value>0.01, *****P-value>0.05 | | | | | | |

| **Supporting File 2**: OM development in regard to the type of HSCT. | | | | | | |
| --- | --- | --- | --- | --- | --- | --- |
| **Parameter** | **Allogeneic**  **(n=134)** | | **Autologous**  **(n=6)** | | **Statistical analysis** | |
|  | **n** | **%** | **n** | **%** | ***X*^2^ (df=1)** | **P-value** |
| **One-week post-HSCT** (n=49) | 46 | 34.3 | 3 | 50.0 | 0.220 | 0.639 |
| **Two-week post-HSCT** (n=36) | 36 | 26.9 | 0 | 0.0 | - | -* |
| **Three-week post-HSCT** (n=13) | 13 | 9.7 | 0 | 0.0 | - | -* |
| * No statistics computed because all patients received allogeneic HSCT. | | | | | | |
